# A clinical herbal prescription Gu-Shu-Kang capsule exerted beneficial effects on the musculoskeletal system of dexamethasone-treated mice by acting on tissue IGF-1 signalling pathway

**DOI:** 10.1080/13880209.2022.2132029

**Published:** 2022-10-21

**Authors:** Xiao-Li Li, Liang Wang, Ming-Chao He, Wen-Xiong Li, Jia-Li Zhang, Yong-Fang Fu, Yan Zhang

**Affiliations:** a School of Medical Instrument and Food Engineering, University of Shanghai for Science and Technology, Shanghai, China; b Department of Geriatric, The Eighth Medical Center of PLA General Hospital, Beijing, China; c Spine Disease Research Institute, Longhua Hospital, Affiliated to Shanghai University of Traditional Chinese Medicine, Shanghai, China; d Department of Trauma, Affiliated Hospital of Shaanxi University of Chinese Medicine, Xianyang, China; e Ministry of Education, Key Laboratory of Theory and Therapy of Muscles and Bones, Shanghai, China

**Keywords:** Bone, muscle, muscle atrophy, myofiber, osteoporosis

## Abstract

**Context:**

Gu-Shu-Kang (GSK) is a clinical traditional Chinese medicine prescription for the treatment of primary osteoporosis.

**Objective:**

This study investigates the protection of GSK against dexamethasone (Dex)-induced disturbance of musculoskeletal system in male mice and to identify the underlying mechanism.

**Materials and methods:**

Male C57BL/6 mice in Dex-treated groups were orally administered (i.g.) with vehicle, low dose (0.38 g/kg), middle dose (0.76 g/kg), or high dose (1.52 g/kg) of GSK for 8 weeks. A control group was designed without any treatment. The quadriceps femoris, tibialis anterior and gastrocnemius were harvested. Molecular expression was determined by RT-PCR and immunoblotting.

**Results:**

Treatment with GSK enhanced weight-loaded swimming time (from 411.7 ± 58.4 s in Dex group to 771.4 ± 87.3 s in GSK-M) and grip strength (from 357.8 ± 23.9 g in Dex group to 880.3 ± 47.6 g in GSK-M). GSK produced a rise in cross-sectional area of myofibers and promoted a switching of glycolytic-to-oxidative myofiber. The administration with GSK affected expression of muscle regulatory factors shown by the down-regulation in MuRF-1 and atrogin-1 and the up-regulation in myogenic differentiation factor (MyoD) and myosin heavy chain (MHC). GSK stimulated tissue IGF-1 signalling pathway (IGF-1R/PI3K/Akt), not only in skeletal muscle but also in bone associated with the amelioration of trabecular bone mineral density and the improvement of osteogenesis.

**Conclusions:**

These findings revealed the potential mechanisms involved in the beneficial effects of Gu-Shu-Kang on musculoskeletal system in mice with challenging to dexamethasone, and this prescription may have applications in management for muscle atrophy and osteoporosis triggered by glucocorticoid.

## Introduction

Glucocorticoid (GC) excess, due to either diseases such as the adrenal gland, stress, ageing, or the chronic administration for immunosuppression, produces adverse effects on the musculoskeletal system shown by the loss in mass of bone and skeletal muscle (Adhikary et al. 2019), consequently leading to osteopenia/osteoporosis and muscle atrophy (Sato et al. 2017). Muscle weakness increases the incidence for falling, which increases the fracture risk when combined with the lower bone mass (Webster et al. 2019). Therefore, given the excess exposure to GC, the health of the musculoskeletal system needs to be regularly monitored in the clinic and might even be managed by medicinal intervention.

The underlying mechanisms for the progressive destructive effects of GC on musculoskeletal system might be closely linked to the insulin growth factor (IGF-1) signalling, which was greatly suppressed in tissues upon to chronic application of GC, followed by the impaired activity of IRS-1-associated PI3K/Akt pathway (Hu et al. 2009; Kim et al. 2016). It was well elucidated that the stimulation on IGF-1 signalling in skeleton could promote bone formation *via* the regulation on osteogenic factors (Chen et al. 2012; Yakar and Isaksson 2016). The activation of IGF-1 signalling in skeletal muscle could result in a hypertrophic effect *via* the inhibition on atrophic factors like MuRF-1 and atrogin-1 (Yoshida et al. 2010; Webster et al. 2019) and the induction on myogenic factors like myogenic differentiation factor (MyoD) and myosin heavy chain (MHC) (Peters et al. 2017). While so far, there are few of research studies reporting the curative effects of potential candidates on GC-induced disruption of musculoskeletal system through locally modulating IGF-1 signalling in tissues.

Gu-Shu-Kang (GSK) capsule, recorded in Chinese Pharmacopoeia elucidating that it is a clinical traditional medicine prescription for treating primary osteoporosis (Li et al. 2001), mainly consists of such traditional Chinese herbs as *Epimedium koreanum* Nakai (Berberidaceae), *Davallia mariesii* T. Moore ex Baker (Davalliacea), *Rehmannia glutinosa* (Gaertn.) DC. (Orobanchaceae), *Astragalus membranaceus* (Fisch.) Bunge (Leguminosae), and *Salvia miltiorrhiza* Bge. (Labiatae). Previous research studies showed that GSK could increase bone mineral density (BMD) and restore trabecular bone microstructure in ovariectomized (OVX) mice (Wang et al. 2018; Li et al. 2019) and rats (Chai et al. 2019), furthermore, we explored the osteopreserve effects of GSK in senile osteoporosis of aged male mice (Li et al. 2020). Our mechanism studies revealed that the modulation of GSK on vitamin D metabolism and calcium homeostasis potentially accounted for its beneficial effects on bone tissue (Li et al. 2019, 2020). Our other research work found that icariin, a bioactive compound isolated from *E. koreanum* that is a monarch drug in GSK prescription, could ameliorate oestrogen deficiency-induced osteoporosis by promoting IGF-I signalling in bone (Zhou et al. 2019, 2021). Moreover, the regulation of icariin on PI3K/Akt pathway was involved in its protection against Parkinson’s disease (Chen et al. 2017). Therefore, we are keen to know whether GSK could ameliorate deterioration of bone and atrophy of muscle associated with GC excess through interfering with IGF-1 signalling.

In this study, the improvements of GSK on the musculoskeletal system, including bone mass and micro-architecture as well as muscular function and muscle fibres, were accurately investigated in dexamethasone-treated mice, an animal model mimicking excess exposure to glucocorticoid, and the underlying action mechanisms behind its therapeutic effects were preliminarily clarified from the respects of osteogenesis and myogenesis through studying the IGF-1 signalling pathway in local tissue.

## Materials and methods

### Source and chemical identification of Gu-Shu-Kang capsule

The GSK capsule (Batch no. 201012) was provided by Konruns Pharmaceutical Co., LTD. (Liaoning, China). The plant name mentioned in this paper has been checked with http://www.theplantlist.org. The two bioactive compounds icariin and naringin in *E. koreanum* and *Davallia mariesii*, respectively, both of which are monarch herbs in GSK, were identified by high-performance liquid chromatography (HPLC) with the separation condition as described previously (Chai et al. 2019). The HPLC profile was displayed and used for quantitative analysis on icariin and naringin as referred in our published paper (Li et al. 2020).

### Animals and treatments

The animals, purchased from GemPharmatech (Jiangsu, China), were housed in environmentally controlled SPF animal facilities, and kept in 22 °C with a 12 h light/dark circle and humidity (45–55%)-controlled condition. All animal procedures were performed in accordance with NIH Guide for Care and Use of Laboratory Animals. The animal study protocol (No. PZSHUTCM200807013) was reviewed and approved by the Animal Care and Use Committee of Longhua Hospital, affiliated to Shanghai University of Traditional Chinese Medicine.

Male 8-week-old C57BL/6 mice were randomly allocated in four groups (*n* = 9), injected daily (i.p.) with dexamethasone (Dex, 5 mg/kg) and co-administrated orally with vehicle, or low (GSK-L, 0.38 g/kg body weight), middle (GSK-M, 0.76 g/kg body weight) or high dose (GSK-H, 1.52 g/kg body weight) of Gu-Shu-Kang by intragastric gavage. The low dose of GSK in mice was equivalent to that used in patients (Shi et al. 2020) and the middle and high doses were set as 2-fold and 4-fold of low dose, respectively, as previously described (Li et al. 2019). A group of age-matched mice without any treatment was designed as control (*n* = 9).

After treatment for 8 weeks, the mice were anaesthetised with combined injection (i.p.) of ketamine (100 mg/kg) and xylazine (10 mg/kg), hence, blood was collected by cardiac puncture and serum was collected and stored at −80 °C for further biochemical analyses. The bilateral tibias and femurs were aseptically removed. The hindlimb muscles including tibialis anterior, quadriceps femoris and gastrocnemius were harvested for a variety of biochemical, histological and molecular analysis.

### Grip strength test

A grip strength metre (Model YLS-13A, China) was used to assess the grip strength of mice limbs. An operator held a mouse gently by the base of the tail, allowing it to grasp the metal bar with its limbs. While the mouse was grasping the metal bar, the operator would flatly pull the mouse backwards by the tail until its grip was lost. The grip strength metre would automatically record the peak force of the gripping in grams (g).

### Weight-loaded swimming test

The performance of endurance in mice was measured by weight-loaded swimming test as previous described (Xia et al. 2015) with minor modification. Briefly, the mice were loaded with lead sheets (5% of mouse body weight) that were attached to the same position of the tail. The weight-loaded mice were individually forced to swim in a columnar swimming pool (50 cm high, 20 cm in diameter and 40 cm deep), which was filled with water at a temperature of 25 ± 1 °C and a depth of 30 cm. The swimming time was recorded until the mice were exhausted.

### Chemistries in serum

The concentrations of calcium and phosphorus in serum were measured by standard colorimetric methods with commercial kits (Wako Pure Chemical Industries Ltd., Osaka, Japan). The serum contents of 25-hydroxyvitamin D (IDS, Boldon, UK) and insulin-like growth factor-1 (Abcam, Boston, MA) were determined with ELISA kits.

### Muscle histomorphology

After embedded in OCT compound (Leica Biosystems, Nußloch, Germany) and frozen in liquid nitrogen-cooled isopentane, the transverse cross-sections (10 μm) of tibialis anterior muscles and gastrocnemius were prepared by a cryostat (CM3050S Leica Biosystems, Nußloch, Germany) at −20 °C and mounted on gelatin-coated glass slides. Haematoxylin and eosin (H&E) staining was performed for observation and assessment of myofibers.

### Immunofluorescence staining

The frozen slides of tibialis anterior underwent anti-dystrophin immunostaining for observation of muscle fibres. The frozen slides of gastrocnemius were incubated with the following antibodies: BA-F8 for MHC type I (1:50), SC-71 for MHC type IIa (1:600), and BF-F3 for MHC type IIb (1:100) (Developmental Studies Hybridoma Bank, Iowa City, IA), subsequently incubated with Alexa Fluor conjugated secondary antibodies 350, 488, and 555 (1:250) (Invitrogen, Carlsbad, CA), followed by staining with regular mounting medium without DAPI (Beyotime, Beijing, China). Fluorescence images were captured by a fluorescence microscope (VS120, Olympus, Tokyo, Japan).

### Micro-CT analysis

The tibia of each animal was scanned to obtain three-dimensional (3D) images and quantitative parameters for trabecular bone within the region between 0.5 mm and 1.5 mm underneath the growth plate, corresponding to 110 slices, at proximal metaphysis. The scanning parameters used were 60 kVp and 417 µA, resulting in a 9.02 μm isotropic voxel size with a high-resolution micro-CT system (SkyScan 1176, Bruker, Germany). The micro-architecture of trabecular bone was reconstructed using the Skyscan Nrecon2 software and the 3D images were viewed with Skyscan CTVol software. The 3D parameters for trabecular bone were expressed as the followings, mean bone mineral density over total volume (BMD/TV), connectivity density (Conn.D), bone volume over total volume (BV/TV), trabecular bone number (Tb.N), trabecular bone thickness (Tb.Th), and trabecular bone separation (Tb.Sp).

### RT-PCR

Total tissue RNA was extracted according to the TRIzol manufacturer’s protocol (Invitrogen, Carlsbad, CA). The cDNAs synthesis was performed by reverse transcription reactions with 3 µg of total RNA using moloney murine leukaemia virus reverse transcriptase (Invitrogen, USA) with oligo dT_(15)_ primers (Fermentas, Burnie, MD). The first strand cDNAs served as the template for the regular PCR with a DNA Engine (ABI). β2M was used as housekeeping gene to determine the relative expression of the target genes. The primers sequence (5′–3′) used in the present study was as the followings: OCN, forward: GACACCATGAGGACCATCTTT; reverse: TAGAGACCACTCCAGCACA; RUNX2, forward; CCCAGCCACCTTTACCTACA; reverse: TATGGAGTGCTGCTGGTCTG; COL-I, forward: CAGACTGGCAACCTCAAGAA; reverse: GGCCAATGTCTAGTCCGAAT; ALP, forward: TGGGCCTGCTCTGTTTCTTC; reverse: CTGAGATTCGTCCCTCGCTG; β2M, forward: ACCGGCCTGTATGCTATCCAGAAA; reverse: ATTTCAATGTGAGGCGGGTGGAAC.

### Immunoblotting

The protein from quadriceps muscle and tibia was extracted by homogenisation in Laemmli buffer supplemented with protease inhibitor cocktail (Roche, Mannheim, Germany). The protein concentration of the lysates was determined using Bradford assay (Beyotime, Beijing, China). Lysates containing 30 µg of protein were separated on SDS-PAGE gel, and transferred onto PVDF membrane (Merck Millipore, Darmstadt, Germany). After saturation with 5% (w/v) non-fat dry milk in TBS and 0.1% (w/v) Tween 20 (TBST), the membranes were incubated with primary antibodies (MHC, MyoD, MuRF-1, atrogin-1, IGF-1R, IRS1, p-PI3K, PI3K, p-Akt, Akt) with dilutions ranging between 1:1000 and 1:2000 at 4 °C overnight (Table 1). After washing with TBST, membranes were incubated with secondary antibodies and enhanced chemiluminescence (ECL) solution (Bio-Rad, Carlsbad, CA). Band intensities were densitometrically assessed by the Lumi-Imager using Lumi-Analyst version 3.10 software (Roche, Mannheim, Germany) and normalized to the expression of GAPDH or β-actin.

**Table 1.** Primary antibodies used for immunoblotting.

Primary antibody	Manufacturer	Code	Specificity
MHC	Abcam	ab91506	Rabbit pAb
MyoD	Santa Cruz Biotechnology	sc-37460	Mouse mAb
MuRF-1	Santa Cruz Biotechnology	sc-398608	Mouse mAb
atrogin-1	Abcam	ab168372	Rabbit mAb
p-PI3K	Abcam	ab182651	Rabbit pAb
PI3K	Abcam	ab191606	Rabbit mAb
p-Akt	Cell Signaling Technology	4060S	Rabbit mAb
Akt	Cell Signaling Technology	4691S	Rabbit mAb
GAPDH	Abcam	ab181602	Rabbit mAb
β-Actin	Sigma	A2228	Mouse mAb
Dystrophin^a^	Abcam	ab15277	Rabbit pAb

^a^Used for immunofluorescence staining

### Statistical analysis

The data from the animal experiments were expressed as mean ± SEM of the values obtained from individual experiments. Bartlett’s test for homogeneity of variances was conducted to evaluate variances associated with each experimental mean. Statistical comparisons between groups were performed by one-way analysis of variance (ANOVA) followed by Tukey *post hoc* test using GraphPad Prism 8.0 (GraphPad software, Inc., San Deigo, CA). A difference of *p* < 0.05 was considered statistically significant.

## Results

### Effects of GSK on chemistries in serum

Dexamethasone treatment did not alter serum level of calcium and phosphorus (Table 2). The serum phosphorus level of Dex-treated mice was markedly decreased (*p* < 0.05) in response to treatment with high dose of GSK. Administration of mice with dexamethasone showed lower concentration (*p* < 0.01) of 25(OH)VD and IGF-1 in serum than those of mice in control group (Table 2). High dose of GSK induced a rise in serum level of 25(OH)VD by 16.3% and of IGF-1 by 59.0% (*p* < 0.05) in a comparison with those in the Dex group.

**Table 2.** Serum chemistry.

	Control	Dex	GSK-L	GSK-M	GSK-H
Calcium (mg/dL)	9.92 ± 0.11	10.38 ± 0.13	10.19 ± 0.22	10.54 ± 0.15	10.18 ± 0.20
Phosphorus (mg/dL)	8.72 ± 0.34	7.96 ± 0.26	8.07 ± 0.42	7.58 ± 0.45	6.54 ± 0.04*
25(OH)VD (ng/mL)	50.66 ± 3.95	37.17 ± 0.86^##^	40.41 ± 1.81	40.71 ± 1.49	43.24 ± 2.49
IGF-1 (ng/mL)	28.92 ± 2.25	17.68 ± 2.48^##^	23.40 ± 2.83	24.90 ± 1.81	28.12 ± 1.52*

The male mice treated with dexamethasone were orally administered with vehicle, or Gu-Shu-Kang at low (GSK-L, 0.38 g/kg), middle (GSK-M, 0.76 g/kg) or high (GSK-H, 1.52 g/kg) doses for 8 weeks. Values were expressed as means ± SEM, *n* = 9. ^##^*p* < 0.01, versus control; *p < 0.05, versus Dex.

### Improvement of GSK on muscular mass, function and strength

**Figure 1.**
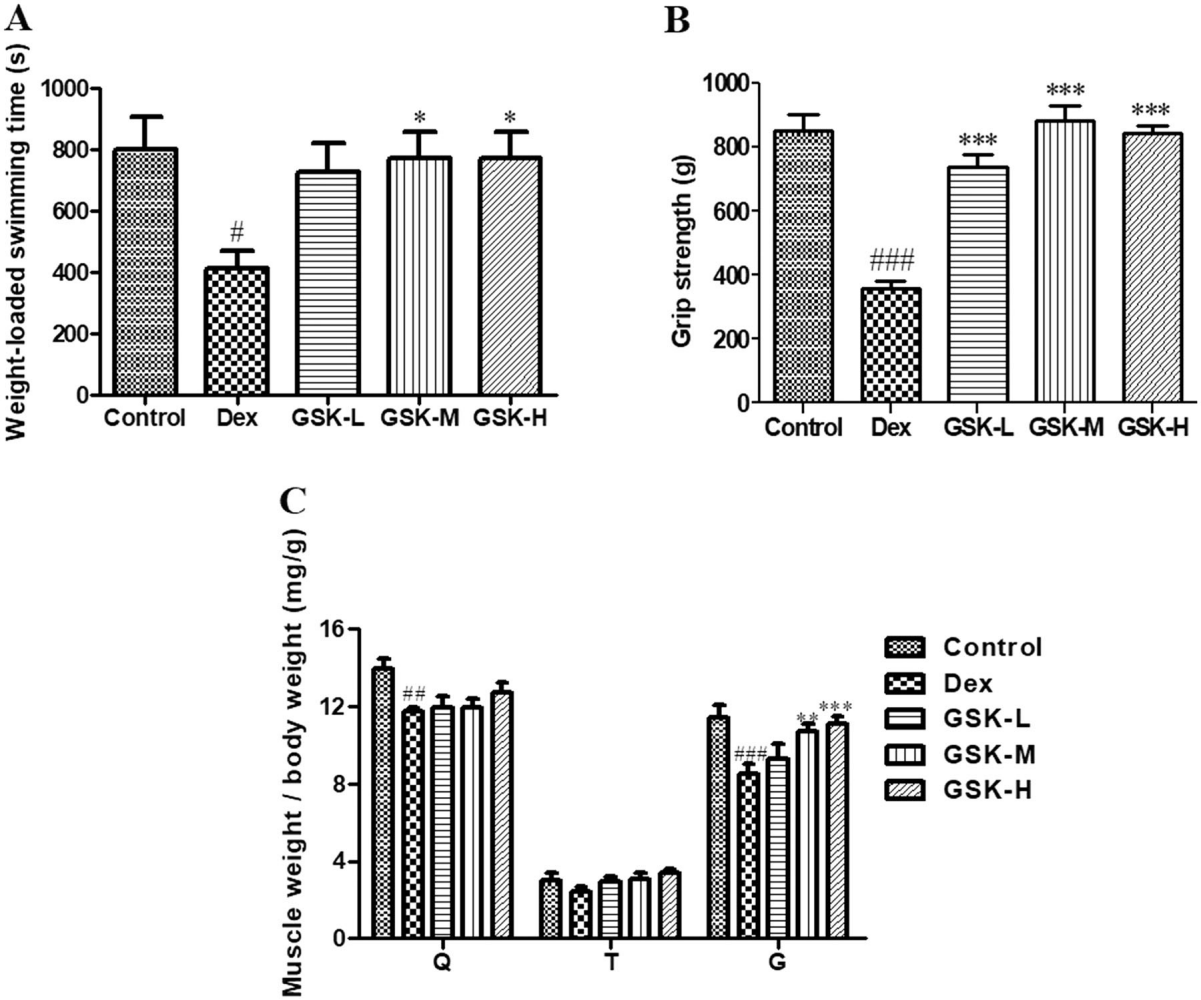
Muscular function and skeletal muscle index. (A) weight-loaded swimming time. (B) Grip strength. (C) Muscle index expressed as the ratio of muscle weight and body weight. Q: quadriceps femoris; T: tibialis anterior; G: gastrocnemius. Values were expressed as means ± SEM, *n* = 9. ^#^*p* < 0.05, ^##^*p* < 0.01, ^###^*p* < 0.001, versus control; *p < 0.05, **p < 0.01, ***p < 0.001, versus Dex.

Treatment of mice with Dex profoundly decreased weight-loaded swimming time (Figure 1(A), *p*< 0.05), grip strength (Figure 1(B), *p*< 0.001) and weight of quadriceps muscle (Figure 1(C), *p*< 0.01) and gastrocnemius (Figure 1(C), *p*< 0.001), suggesting a significant reduction in muscular function, strength and mass, respectively. Administration of GSK for 8 weeks showed much higher in weight-loaded swimming time (*p* < 0.05), grip strength (*p* < 0.001) and gastrocnemius weight (*p* < 0.01) in groups of middle dose and high dose than those of the Dex-treated group. The weight of quadriceps muscle and tibialis anterior did not show statistical difference between GSK-treated groups and vehicle-treated Dex group.

### GSK increased myofiber size of Dex-treated mice

**Figure 2.**
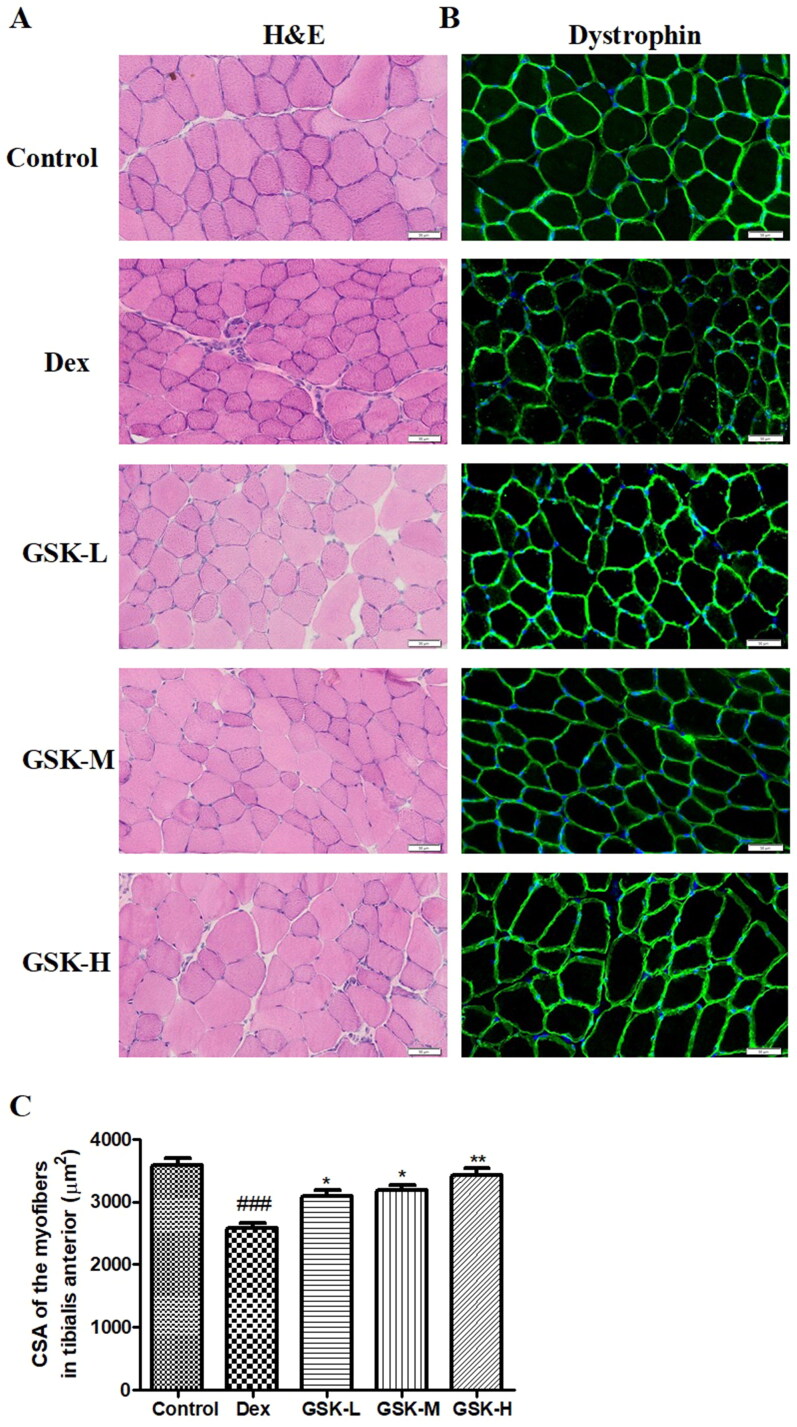
Histomorphology of muscle fibres and cross-sectional area of myofibers in tibialis anterior. (A) Haematoxylin and eosin (H&E) staining. (B) Anti-dystrophin immunostaining. (C) Quantitative data on cross-sectional area of myofibers. The colour with deep blue (A) and the positive staining with green fluorescence (B) indicate the cross section of myofibers. Values were expressed as means ± SEM, *n* = 9. ^###^*p* < 0.001, versus control; *p < 0.05, **p < 0.01, versus Dex.

The results from the H&E staining (Figure 2(A)) and the immunofluoresence staining on dystrophin (Figure 2(B)) showed the significant drop (*p* < 0.001) in the cross-sectional area (CSA) of tibialis anterior of Dex-treated mice (Figure 2(C)), indicating the exposure to dexamethasone triggered injuries in skeletal muscle. The quantitative dada clearly displayed that the myofiber size of tibialis anterior in GSK-treated mice was statistically increased (*p* < 0.05) with dose-dependent manner in a comparison with that in the Dex group.

### GSK improved composition of fibre types of Dex-treated mice

**Figure 3.**
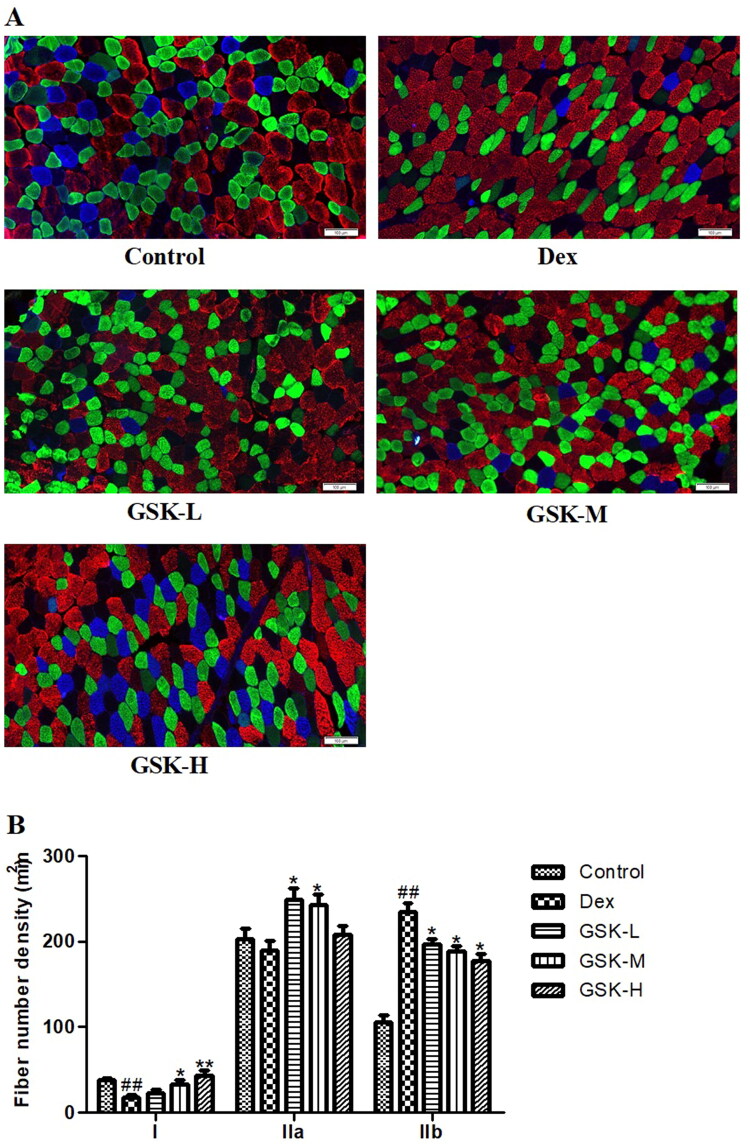
Distribution and composition of myofiber types in gastrocnemius. Blue, type I; Green, type IIa; Red, type IIb. (A) Images. (B) Quantitative data on the density of respective muscle fibre type. Values were expressed as means ± SEM, *n* = 9. ^##^*p* < 0.01, versus control; *p < 0.05, **p < 0.01, versus Dex.

The beneficial effect of GSK on skeletal muscle was also illustrated by the distribution in fibre type of mice gastrocnemius with immunofluorescence staining which indicated type I (blue), type IIa (green) and type IIb (red) fibres (Figure 3(A)). Treatment with dexamethasone remarkably reduced the density of type I fibres (*p* < 0.01) and enhanced the density of type IIb fibres (*p* < 0.01) as well as decreased type IIa fibres density by 6.9% (Figure 3(B)). GSK dose-dependently enhanced the density of type I fibres with statistical significance in the GSK-M group (*p* < 0.05) and the GSK-H group (*p* < 0.01), and produced a significant decrease in density of type IIb fibres in all groups (*p* < 0.05), as compared to those of mice with exposure to dexamethasone treatment alone. Additionally, both low dose and middle dose of GSK induced an elevation in distribution of type IIa fibres of mice gastrocnemius (*p* < 0.05).

### Regulation of GSK on muscle regulatory factors (MRFs) in quadriceps muscle

**Figure 4.**
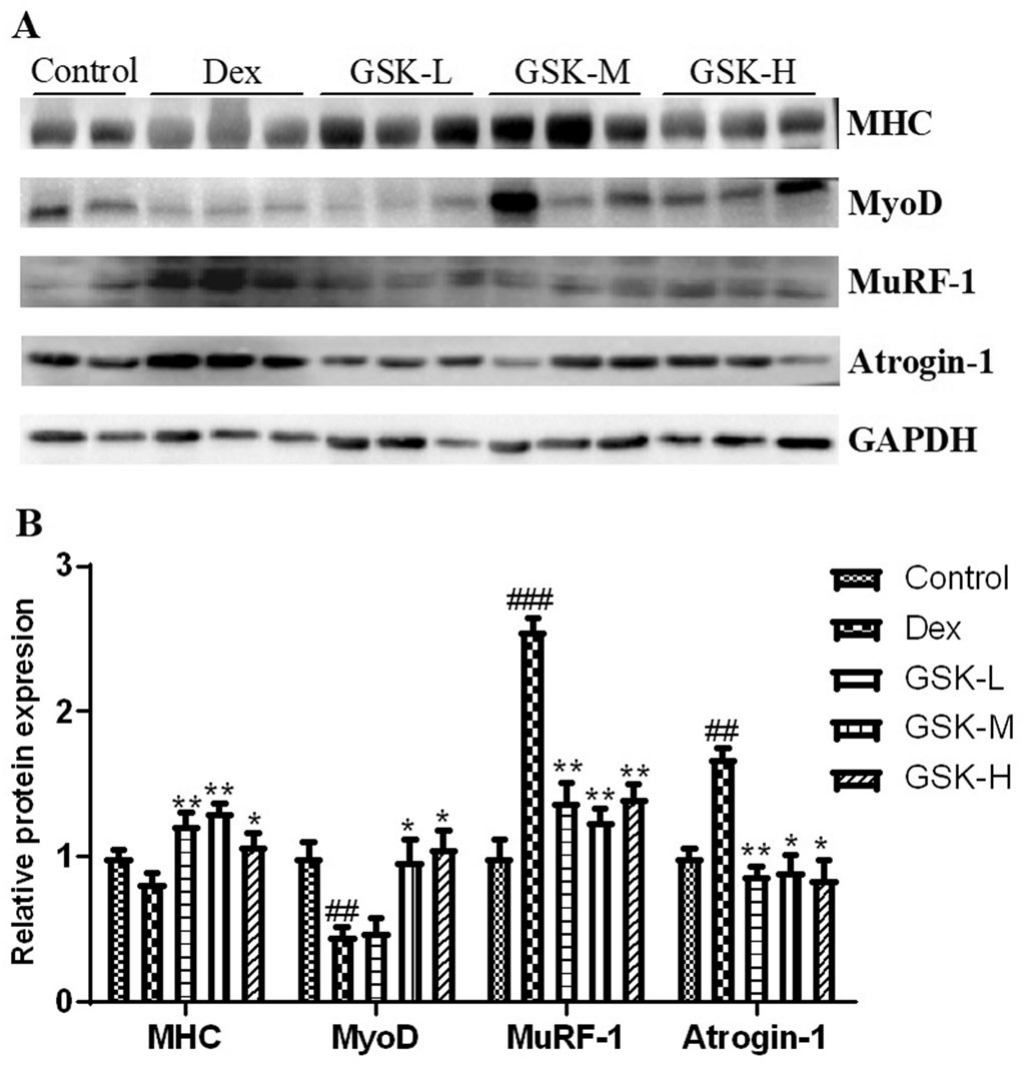
Protein expression of muscle regulatory factors (MRFs) in quadriceps femoris. (A) Western blotting. (B) The quantitative data on target proteins. Values were expressed as means ± SEM, *n* = 9. ^##^*p* < 0.01, ^###^*p* < 0.001, versus control; *p < 0.05, **p < 0.01, versus Dex.

The protein expression of muscle regulatory factors in quadriceps femoris was detected (Figure 4(A)). Dex treatment significantly up-regulated expression of MuRF-1 (*p* < 0.001) and atrogin-1 (*p* < 0.01), and down-regulated expression of MyoD (*p* < 0.01) as well as lowered MHC expression by 18% (Figure 4(B)), as compared to those of the control group. The protein expression of MuRF-1 (*p* < 0.01) and atrogin-1 (*p* < 0.05) was dramatically decreased in quadriceps muscle of Dex-treated mice after administration with GSK. The MHC expression was much higher in GSK-treated groups (*p* < 0.05) than that of the Dex group, moreover, both middle dose and high dose of GSK increased MyoD expression by more than 1-fold (*p* < 0.05) in a comparison with that of Dex-treated mice.

### Regulation of GSK on expression of factors involved in IGF-1 signalling in quadriceps muscle

**Figure 5.**
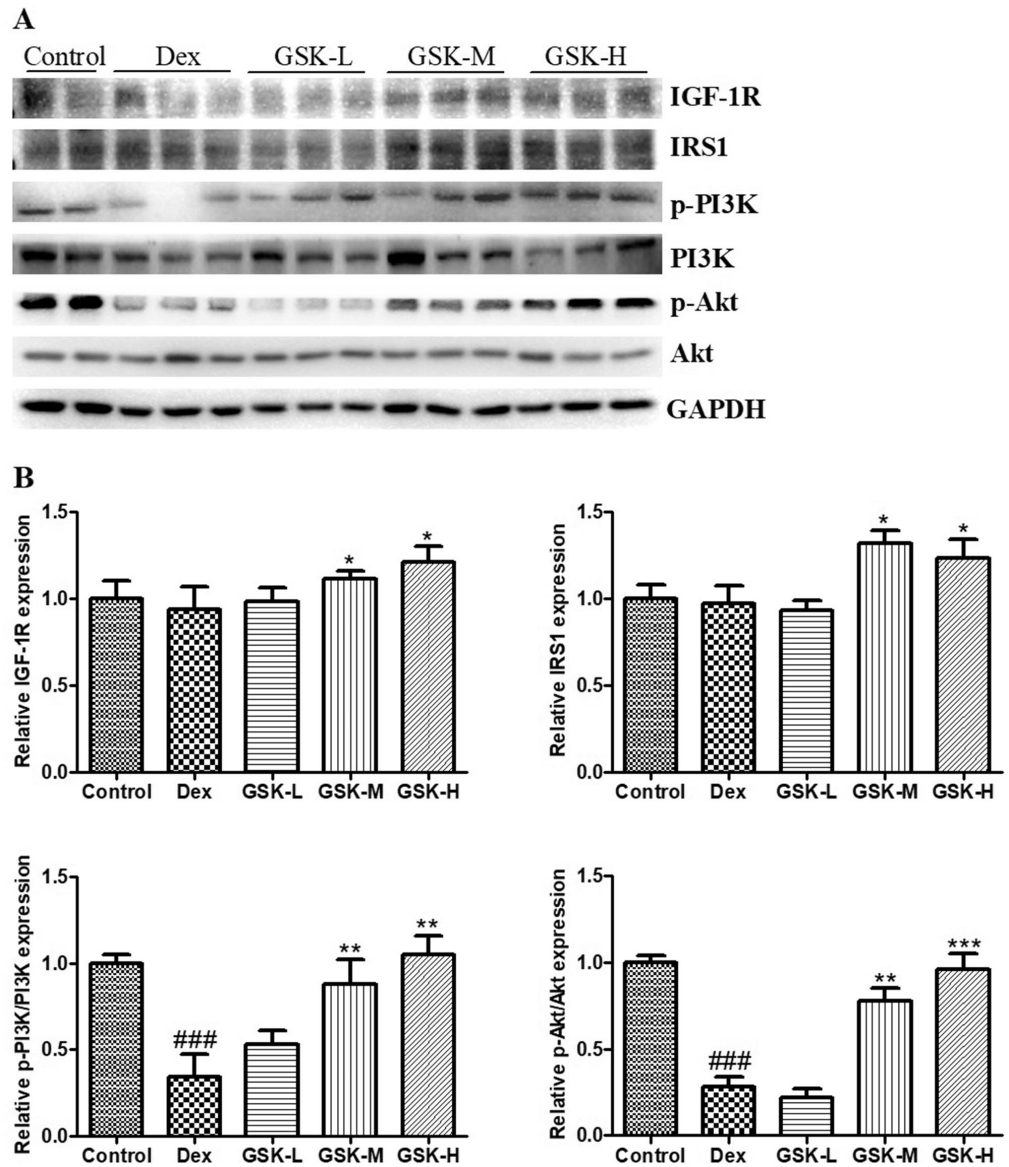
IGF-1 signalling and the protein expression of factors in this pathway in quadriceps femoris. (A) Western blotting. (B) The quantitative data on target proteins. Values were expressed as means ± SEM, *n* = 9. ^###^*p* < 0.001, versus control; * *p* < 0.05, **p < 0.01, ***p < 0.001, versus Dex.

The protein expression of factors involved in IGF-1 signalling in quadriceps muscle of mice was further determined (Figure 5(A)). Treatment of mice with dexamethasone induced a significant down-regulation (Figure 5(B), *p*< 0.001) in expression ratio of p-PI3K/PI3K and p-Akt/Akt, but not in expression of either IGF-1R or IRS1. The GSK treatment with middle dose and high dose markedly affected the expression level of protein components in IGF-1 signalling pathway (Figure 5(B)), as demonstrated by the up-regulation of IGF-1R (*p* < 0.05), IRS1 (*p* < 0.05), ratio of p-PI3K/PI3K (*p* < 0.01) and p-Akt/Akt (*p* < 0.01).

### Effect of GSK on trabecular bone at the proximal tibial metaphysis

**Figure 6.**
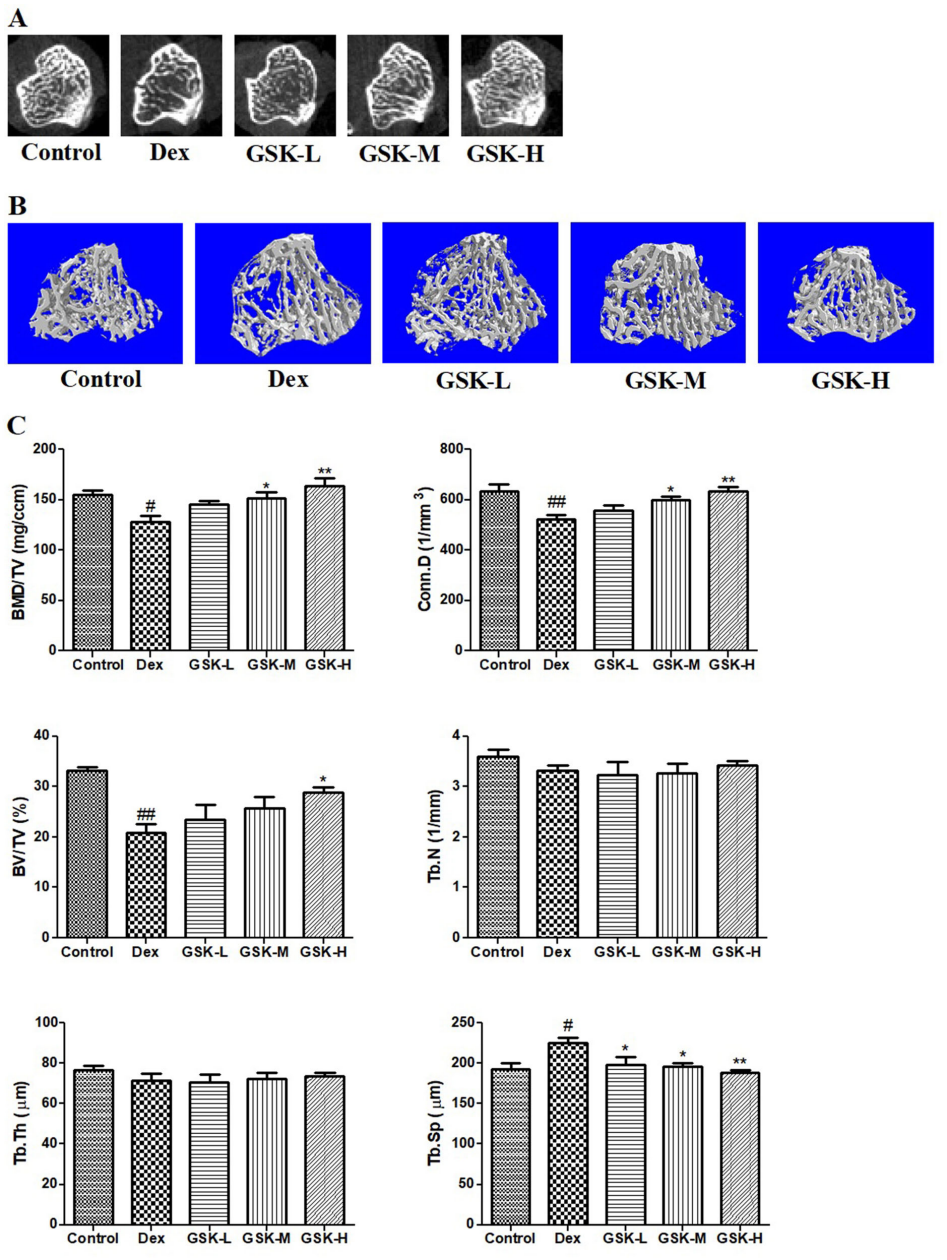
Trabecular bone properties measured by micro-CT at proximal metaphysis of tibia. (A) 2D images. (B) Reconstructed 3D images. (C) The quantitative parameters of trabecular bone including bone mineral density over total volume (BMD/TV), connectivity density (Conn.D), bone volume over total volume (BV/TV), trabecular bone number (Tb.N), trabecular bone thickness (Tb.Th), and trabecular bone separation (Tb.Sp). Values were expressed as means ± SEM, *n* = 9. ^#^*p* < 0.05, ^##^*p* < 0.01, versus control; *p < 0.05, **p < 0.01, versus Dex.

The profiles of two-dimensional (2D, Figure 6(A)) and 3D (Figure 6(B)) images obviously displayed the loss of trabecular bone mass and the breakage of cancellous bone at proximal metaphysis of tibia of Dex-treated mice. The quantitative data showed a decline in trabecular bone mineral density (BMD/TV, *p* < 0.05), connectivity density (Conn.D, *p* < 0.01), and trabecular bone volume (BV/TV, *p* < 0.01), as well as a rise in trabecular bone separation (Tb.Sp, *p* < 0.05) in the Dex group as compared to those of the control group (Figure 6(C)). The values of BMD/TV and Conn.D in mice treated with low dose of GSK were increased by 13.3% and 7.1%, respectively, in a comparison with the Dex group. GSK treatment in GSK-M (*p* < 0.05) and GSK-H (*p* < 0.01) groups dose-dependently produced a dramatic elevation in BMD/TV and Conn.D and a marked drop in Tb.Sp at the proximal tibial end of mice.

### Effect of GSK on mRNA expression of osteogenesis factors in bone of mice

**Figure 7.**
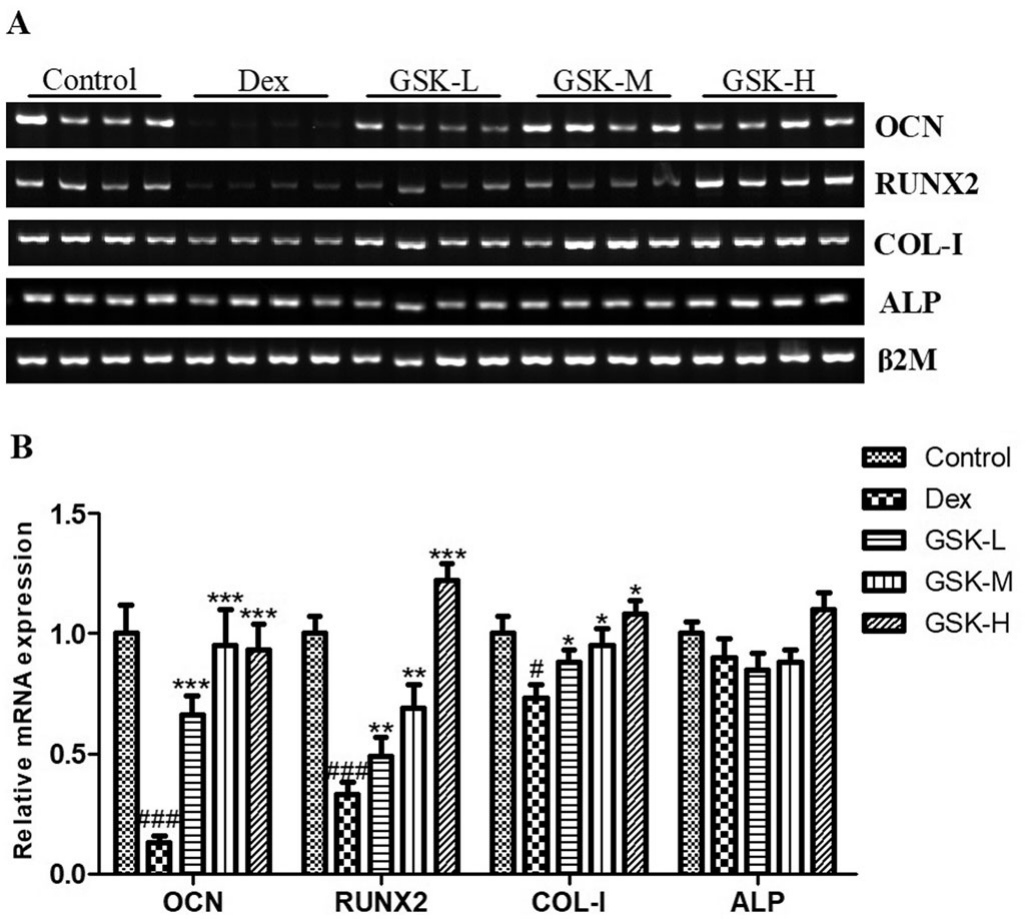
mRNA expression of osteogenic factors in femur. (A) PCR bands of osteocalcin (OCN), runt-related transcription factor 2 (RUNX2), type I collagen (COL-I) and alkaline phosphatase (ALP). (B) The quantitative data on target genes. Values were expressed as means ± SEM, *n* = 9. ^#^*p* < 0.05, ^###^*p* < 0.001, versus control; *p < 0.05, **p < 0.01, ***p < 0.001, versus Dex.

The quantitative analysis on PCR products in mouse femur (Figure 7(A)) found that treatment with GSK dramatically up-regulated the mRNA expression of osteocalcin (OCN, *p* < 0.001), and dose-dependently raised the mRNA expression of RUNX2 (*p* < 0.01) and type I collogen (COL-I, *p* < 0.05), as compared to those of mice treated with dexamethasone only (Figure 7(B)). However, gene expression of alkaline phosphatase (ALP) was not statistically different among groups. These results partially demonstrated the potential stimulatory effects of GSK on osteogenesis in mice after challenging to the induction with dexamethasone.

### Regulation of GSK on protein expression of factors involved in IGF-1 signalling in bone of mice

**Figure 8.**
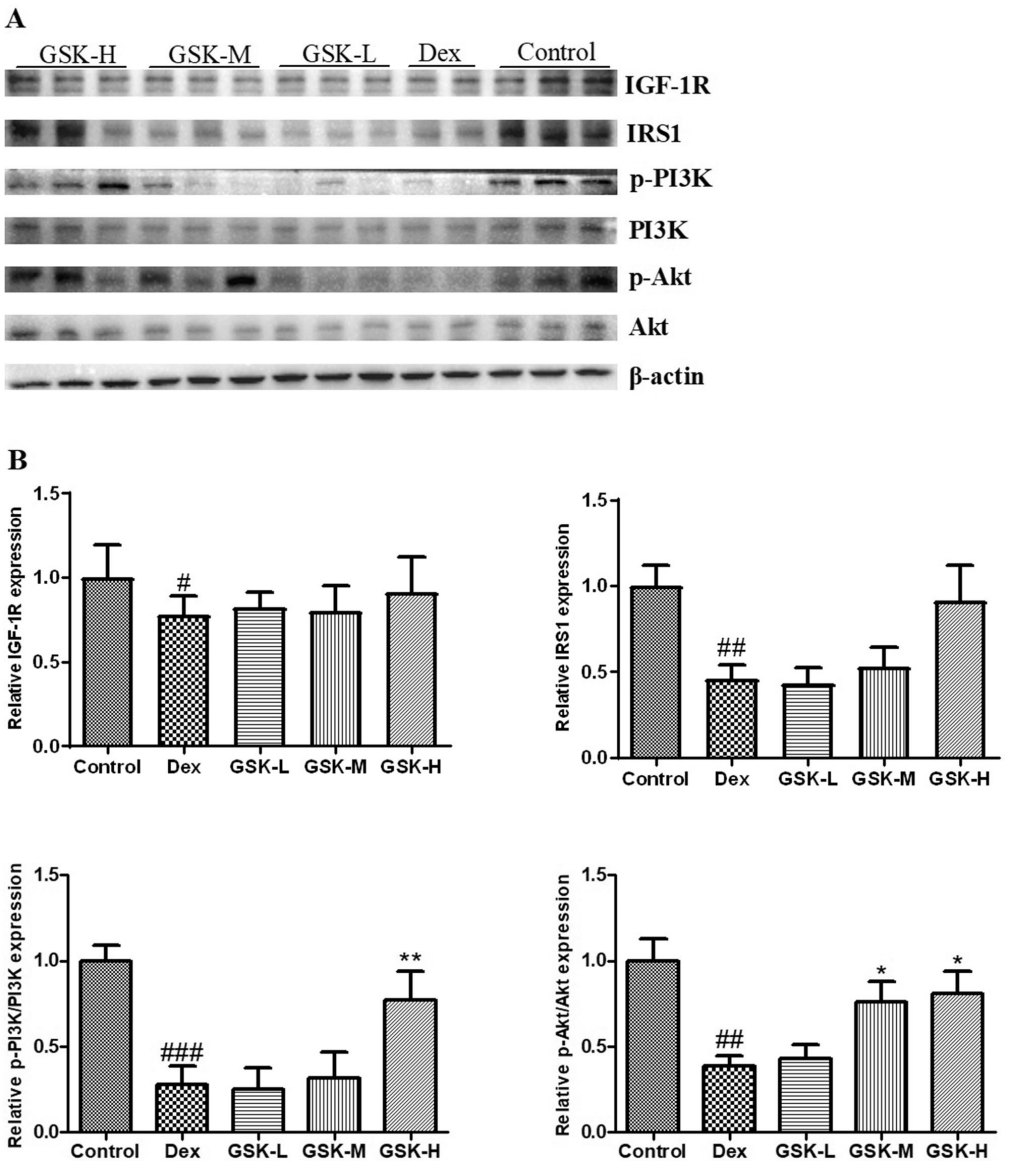
IGF-1 signalling and the protein expression of factors in this pathway in tibia. (A) Western blotting. (B) The quantitative data on target proteins. Values were expressed as means ± SEM, *n* = 9. ^#^*p* < 0.05, ^##^*p* < 0.01, ^###^*p* < 0.001, versus control; *p < 0.05, **p < 0.01, versus Dex.

To clarify the beneficial effects of GSK on bone tissue and osteogenesis, the IGF-1 signalling was also evaluated (Figure 8(A)) in mouse tibia since it displayed actions on this pathway in skeletal muscle in this study. The dexamethasone treatment played inhibitory effects (Figure 8(B)) on the expression of IGF-1R (*p* < 0.05), IRS1 (*p* < 0.01), p-PI3K/PI3K (*p* < 0.001), and p-Akt/Akt (*p* < 0.01). GSK with high dose enhanced the expression ratio of p-PI3K/PI3K (*p* < 0.01), and both middle dose and high dose of GSK raised the expression ratio of p-Akt/Akt (*p* < 0.05), as compared to those of mice treated with dexamethasone alone.

## Discussion

The therapeutic effects of glucocorticoids (GCs) have been known and used for a long time, given that GCs represent the most essential and frequently used class of anti-inflammatory drugs (Schäcke et al. 2002). While along with the chronic application of GCs, the numerous side effects have been found in local tissues such as bone (osteoporosis) and skeletal muscle (muscle atrophy). Currently the medical therapeutic options that reverse the tissue-specific consequences due to circulatory GC excess are limited (Morgan et al. 2016). In this study, we are trying to delineate the protective effects of Gu-Shu-Kang (GSK) capsule, a TCM prescription for the treatment of primary osteoporosis, against GC-elicited adverse effects on the musculoskeletal system as well as to investigate the underlying mechanisms in dexamethasone (Dex)-treated mice.

The present study uncovered that treatment with GSK abrogated Dex-mediated adverse effects on muscle mass and muscular function as demonstrated by the enhancement in weight-loaded swimming time and grip strength, thus, this *in vivo* study could be recognized as the first to provide research data revealing the therapeutic efficacy of GSK on muscle atrophy induced by Dex. Further H&E staining and immunostaining on dystrophin were carried out to assess the alterations of myofibers from the view of microstructure of skeletal muscle. Paralleled by the changes in muscle function, treatment of mice with GSK mitigated the deleterious effect of Dex on the cross-sectional area of myofibers, fully reflecting the beneficial effects of GSK on maintaining musculature health.

The distribution and composition of myofibers directly influence physiological and biochemical properties of skeletal muscle (Meng et al. 2020). The distinct myofiber types are normally defined by myosin heavy chain (MHC) isoforms clarified by specific staining, including type I (slow-twitch oxidative), type IIa (fast-twitch oxidative), and type IIb (fast-twitch glycolytic). This study found that Dex remarkably decreased the density of type I and modestly reduced the density of type IIa muscle fibres as well as stimulated a distribution of type IIb muscle fibres, suggesting an inhibition on switching of glycolytic-to-oxidative (GTO) fibre type. The myofiber transition from glycolytic to oxidative, which is a classic effect of exercise (Badin et al. 2016), could be evoked by traditional functional food *Lycium barbarum* L. (Solanaceae) (Meng et al. 2020) and functional nutritional component resveratrol (Jiang et al. 2019), both of which were reported to exert mimetic effects on skeletal muscles in a manner similar to exercise. Intriguingly, treatment with GSK increased the number of oxidative type myofiber by promoting a GTO switch, paralleled by the increase in muscle strength and endurance in this study. Overall, our study revealed a unique role of GSK in the maintenance of muscle fibre-type distribution with a classic exercise-like effect in locomotory muscles of the hindlimb.

It is well elucidated that the ubiquitin–proteasome system plays crucial effect in regulating musculature protein metabolism, and a reduction in muscle mass is associated with the up-regulation of ubiquitin ligases (Bonaldo and Sandri 2013). Two muscle-specific ubiquitin E3 ligases atrogin-1 and MuRF-1 have been identified to be strongly up-regulated in muscle atrophy models (Yoshida et al. 2013). Functionally, atrogin-1-dependent ubiquitination promotes the degradation of MyoD, a key transcription factor regulating myoblast differentiation, and MuRF-1 acts on the substrate sarcomeric protein MHC to accelerate muscle proteolysis (Bonaldo and Sandri 2013). This study delineated that GSK successfully relieved the Dex-induced up-regulation of atrogin-1 and MuRF-1, resulting in an elevation in protein expression of MyoD and MHC, partially explaining an ameliorative effect of GSK on muscle atrophy through affecting muscle protein synthesis and degradation.

Besides beneficial effects of GSK on skeletal muscle in this study, the protective effects of GSK against Dex-induced defects in bone were also illustrated. As expected, the mice treated with dexamethasone showed characteristic changes in bone tissue, so called glucocorticoid-induced osteoporosis (GIOP), as revealed by the loss of trabecular bone mineral density and the deterioration of trabecular bone network structure. The present study re-confirmed the osteoprotective effects of GSK in GIOP mice model following our early studies elaborating its therapeutic effects on primary osteoporosis in oestrogen-deficiency mice (Li et al. 2019) and aged mice (Li et al. 2020). Additionally, the regulatory effects of GSK on osteogenesis were reflected by the up-regulation in mRNA expression of RUNX2, OCN and COL-I in bone of mice with exposure to Dex, which was consistent with the *in vitro* study showing its stimulatory effect on osteoblastogenesis (Wang et al. 2018). Collectively, this *in vivo* study discovered the bone-sparing efficacy of GSK in dexamethasone-treated mice and shed light on the potential implications in musculoskeletal disorders arising from the GCs effects.

The reduction in serum phosphorus (P) level was noted in both our early study (Li et al. 2019) and the present study, suggesting a potential affection of GSK at high dose on phosphorus homeostasis. We will try to clarify the underlying actions of GSK on phosphorus metabolism through further studies via assessing such as the absorption and reabsorption of P in intestine and in kidney, respectively, the relevant transporters for transferring P, and the hormones like fibroblast growth factor-23 and parathyroid hormone which play central role in controlling P metabolism.

When further exploring the underlying mechanism behind the beneficial effects of GSK on musculoskeletal system under dexamethasone induction, the IGF-1 signalling was evaluated in muscle and bone, since this pathway actively participates in myogenesis (muscle mass entity and strength development) (Ahmad et al. 2020; Yoshida and Delafontaine 2020) and osteogenesis (bone mass and bone accrual) (Courtland et al. 2011; Xian et al. 2012), and low serum IGF-1 level was associated with low handgrip strength, poor physical performance and low bone mineral density (Mohamad and Khater 2015; Ahmad et al. 2020). In this study GSK treatment increased serum level of IGF-1 in a dose-dependent manner in Dex-treated mice, suggesting a potential role of GSK in promoting IGF-1 production in circulation. Given the positive effects of IGF-1 produced locally on tissue development like skeletal muscle hypertrophy (Sculthorpe et al. 2012) and bone regeneration (Zhang et al. 2020), whether the IGF-1 signalling pathway in muscle and bone could be affected by GSK need to be further clarified.

IGF-1R is an IGF-1 receptor with a transmembrane location that activates downstream PI3K/Akt signalling, and insulin receptor substrate (IRS)-1 could prolong IGF-1R activity on the cell surface and ensure sustained IGF-1 bioactivity by delaying IGF-1R endocytosis (Yoneyama et al. 2018). Our study clearly revealed that GSK treatment could increase the expression of IGF-1R and IRS-1 in muscle and exert an induction on the PI3K/Akt pathway in bone and muscle, which accounted for the management of GSK on musculoskeletal system in mice treated with Dex. Furthermore, the regulation of GSK on IGF-1 signalling might contribute to its inhibition on atrogin-1 and MuRF-1 in muscle and its stimulation on expression of osteogenic factors in bone, as the activation of IGF-1R/PI3K/Akt pathway could lead to a rise in protein synthesis and a decrease in ubiquitin ligases activity in muscle (Moarbes et al. 2019; Yoshida and Delafontaine 2020) and promote osteogenesis-related genes transcription in bone tissue (Zhang et al. 2020).

A recent study reported that the extract of *E. koreanum*, recognized as monarch herb in GSK, stimulated C2C12 myotube hypertrophy by activating key components like IGF-1R within the IGF-1 signal pathway (Lin et al. 2021), which was also involved in the regulatory effects of icariin, the bioactive flavonoid in *E. koreanum*, in astrocytes (Zhang et al. 2021) and in myoblasts (Lin et al. 2021) as well as in the protection from oestrogen deficiency-induced bone loss in OVX rats (Zhou et al. 2021). Thus, it was assumed that *E. koreanum* and its active ingredient icariin might, at least partially, could contribute to the management of GSK on musculoskeletal system under Dex exposure in this study. Since the GSK prescription is a mixture of multiple components, the other active materials will be figured out in further study. In addition, the existence and content of the compounds and/or the metabolites in serum need to be identified and the respective activity on musculoskeletal system would be evaluated by *in vivo* and *in vitro* experimental approaches. Attractively, the investigations on the clinical samples using serum of patients after treatment with GSK prescription will enrich and strengthen the evidences for understanding the bioactive components and the underlying action mechanisms.

## Conclusions

Taken together, this study re-confirmed the therapeutic effects of GSK on osteoporosis in a murine model established by glucocorticoids excess, and disclosed that long-term treatment of mice with GSK could improve muscular function and increase muscle mass as well as effectively regulate myofiber size and switch of myofiber types. The action mechanism underlying these effects might be attributed to GSK-triggered IGF-1 signalling, which was involved in its promotion on osteogenesis and myogenesis in dexamethasone-treated mice. It is hard to conclude the ‘best dose’ of GSK based on our current study. While, for the assessment on tissue phenotypes, it might be appropriate to say that the low and middle doses are within the effective dose range, and we should pay more attention to the safety of GSK in high dose given the potential influence on phosphorus metabolism which needs to be further explored. Collectively, GSK exerted beneficial effects on musculoskeletal system of mice with exposure to dexamethasone, suggesting a novel clinical intervention strategy for skeletal muscle atrophy and wasting, even sarcopenia in people associated with glucocorticoids excess and/or ageing.
